# Effects of a Six-Month Physical Activity Program on Health Risk Factors and Body Composition Among Overweight and Obese Middle-Aged Adults

**DOI:** 10.3390/healthcare12212140

**Published:** 2024-10-28

**Authors:** Artur Białkowski, Piotr Soszyński, Jarosław Pinkas, Janusz Ostrowski, Urszula Religioni

**Affiliations:** 1 School of Public Health, Centre of Postgraduate Medical Education of Warsaw, 01-813 Warsaw, Poland; 2 Medicover Sp. z o.o., 00-807 Warsaw, Poland

**Keywords:** risk factors, overweight, obesity, primary prevention, physical activity, body mass composition

## Abstract

**Introduction.** Physical activity is vital for preventing and managing lifestyle-related diseases, which pose significant health and socio-economic challenges. This study aimed to evaluate the effects of a six-month supervised physical activity program on health risk factors and body composition in middle-aged individuals with overweight or obesity. **Methods.** The study involved 166 men and women aged 30 to 65 years, with a body mass index (BMI) ranging from 26 to 35 and moderate to severe health risks. Participants of the study were engaged in a six-month physical activity training program designed in accordance with World Health Organization guidelines. Comprehensive assessments were performed at baseline and after the intervention, including measurements of various anthropometric and body composition parameters, as well as evaluations of functional performance. Fitness tests were also conducted to assess participants’ physical capacity and to monitor improvements throughout the training period. **Results.** Baseline measures of body mass composition and age demonstrated a strong positive correlation with visceral fat rating (VFR) in both men (r = 0.364, *p* < 0.001) and women (r = 0.420, *p* = 0.002). Following the six-month training program, participants exhibited an average improvement of 30% (*p* < 0.001) in their Cooper endurance test results. The physical activity intervention positively impacted body mass index (BMI) and various body composition metrics, including fat mass, VFR, and muscle mass, across all participants and most subgroups (gender, BMI, and age). In males, training at higher maximum heart rate (HRmax) zones significantly contributed to a reduction in the percentage of fat mass (80–89% HRmax) and an increase in the percentage of muscle mass (70–79% and 80–89% HRmax). **Conclusions.** The study indicates that a six-month supervised physical activity program significantly improves health risk factors and body composition (visceral fat reduction and improvement in percent of fat and muscle mass) among middle-aged individuals with overweight or obesity. Therefore, we advocate for the integration of structured physical activity interventions into healthcare practices to effectively enhance health outcomes in this population.

## 1. Introduction

Physical activity is widely recognized for its dual role in both the prevention and treatment of numerous health conditions. As an effective intervention, it has been demonstrated to mitigate the risk of a broad spectrum of diseases, particularly non-communicable diseases (NCDs), such as cardiovascular disorders, type 2 diabetes, obesity, and certain cancers [1,2]. These lifestyle-related diseases represent a significant global health challenge, contributing to substantial morbidity and mortality, as well as imposing significant socio-economic burdens on healthcare systems and societies. The preventive effects of physical activity are well-documented, positioning it as a critical factor in reducing the incidence of these conditions and addressing the rising epidemic of NCDs worldwide [3,4].

In addition to its preventive function, physical activity plays a therapeutic role in individuals already affected by chronic health conditions. For those with overweight or obesity, metabolic disorders, mental health conditions, musculoskeletal disorders, and a variety of other pathologies, regular physical activity has been shown to facilitate recovery and, in some cases, slow or even halt the progression of the underlying disease [5,6]. Exercise induces physiological adaptations across multiple systems, improving cardiovascular function, enhancing metabolic health, promoting mental well-being, and strengthening the musculoskeletal system. These benefits make physical activity a key non-pharmacological intervention in disease management and rehabilitation [1].

Importantly, physical activity occupies a central position in the continuum of disease prevention and management, operating at all three levels of prevention [7,8]. In primary prevention, it acts to avert the onset of disease by maintaining optimal health. In secondary prevention, physical activity supports early intervention and disease detection, thereby reducing the progression of nascent health conditions. In tertiary prevention, it is integral to rehabilitation efforts, aiding in the recovery from illness, preventing complications, and minimizing the risk of recurrence. Consequently, physical activity is a fundamental element in public health strategies aimed at curbing the global burden of chronic diseases and enhancing population health outcomes.

Physical activity levels tend to increase from childhood through early adulthood, but subsequently decline with advancing age. Although men generally maintain higher activity levels than women across all age groups, the rate of decline with age is comparable between genders, with noticeable reductions beginning as early as the fifth decade of life [9]. Consequently, such reduced levels of physical activity may contribute to an overall increase in health risks associated with aging, thereby heightening the likelihood of developing metabolic disorders and other chronic diseases [10,11]. Engaging in physical activity and sport can partially mitigate these negative health changes and improve individuals’ risk profiles. However, recent global studies indicate that levels of physical activity remain relatively low and are continuing to decline [12]. This observation has been confirmed by us in a recent review concerning insufficient physical activity in the Polish population [13].

Body composition is a critical factor that undergoes significant changes with aging and adversely impacts health risk. In middle-aged adults, the accumulation of visceral fat, increased adipose tissue, and the loss of muscle mass and strength collectively contribute to an elevated health risk [14]. The results of various studies suggest that physical activity may, at least partially, reverse the negative changes in body composition associated with obesity and aging. These findings highlight the potential of structured physical training to improve health outcomes by promoting the maintenance of lean muscle mass and reducing visceral fat, both of which are critical for mitigating the risks of metabolic and cardiovascular diseases. However, while the positive effects of physical activity on body composition are well-documented, further research is essential to determine the most effective types, intensities, and durations of exercise interventions. Additionally, understanding how to tailor these interventions to different populations, taking into account factors such as age, gender, and baseline fitness levels, is crucial for maximizing their impact [15,16].

The aim of this study was to evaluate the impact of a six-month supervised physical activity program, implemented as a sole intervention in real-life settings, on selected health risk factors and body composition in a middle-aged working population with overweight or obesity and elevated health risk. The research hypothesis is that a six-month supervised physical activity program will significantly improve health risk factors and body composition in the study population. This study introduces a novel perspective by examining the effects of a six-month supervised physical activity program on health risk factors and body composition in middle-aged individuals with overweight or obesity, focusing on visceral fat reduction and muscle mass gain. Unlike previous research, it explores the impact of sustained, real-life interventions, providing new insights into how varying exercise intensities influence specific body composition metrics across different subgroups. This study’s originality lies in its detailed analysis of heart rate zones and their correlation with improved health outcomes, which has not been extensively studied. The findings have significant implications for public health, offering a scalable, evidence-based model for integrating structured physical activity into healthcare practices to combat non-communicable diseases. 

## 2. Materials and Methods

### Characteristics of the Study Group

The study included 166 professionally active men and women with overweight or stage I obesity with moderate to severe health risk, aged 30–65.

Inclusion criteria for the study included the following:

- age > 30 years, stratified into 3 age groups: 30–39 years, 40–49 years, and >50 years,
- values documented in medical records during last 12 months before inclusion in the study:
  ○ BMI ≥ 26, but not higher than 35, stratified into 2 groups: BMI 26–29.9 (overweight) and 30–35 (obesity):
  and at least one of health risk factors:
  ○ total serum cholesterol concentration ≥ 220 mg/dL,
  ○ fasting serum glucose concentration ≥ 100 mg/dL,
  ○ last blood pressure measurement > 140/90 mmHg,
- lack of regular physical activity in the 12 months before entering the study.

Exclusion criteria from the study included the following:

- diagnosis of a chronic disease that limits the possibility of long-term training, such as the following conditions:
  ○ advanced diabetes,
  ○ arterial hypertension that is not adequately controlled according to the doctor,
  ○ chronic ischemic heart disease,
  ○ heart failure,
  ○ asthma, chronic obstructive pulmonary disease (COPD), and other chronic lung diseases,
  ○ other chronic diseases that could adversely affect the ability to participate in the study or pose a threat to the study participant.
- Cancer, undergoing treatment or diagnosed within 5 years preceding participation in the study,
- pregnancy,
- other conditions that prevent moderate or intense physical activity, e.g., limitations in the musculoskeletal system,
- all other health problems and disorders that, in the opinion of the doctor, prevented participation in the study.

## 3. Research Methods

The study was conducted between March and October 2023 and involved a structured physical activity program consisting of multiple stages designed to assess the impact of exercise on participants’ health outcomes.

### 3.1. Recruitment Stage

1. **Initial Qualification**: Participants were screened for eligibility based on predefined inclusion and exclusion criteria, which were determined using data from their electronic medical records.
2. **Invitation Process**: A trained nurse conducted phone invitations to those initially qualified for the study.
3. **Initial Visits**: Participants attended three initial assessment visits:
  ○ **Visit 1**: Participants met with a family medicine or internal medicine specialist trained in lifestyle medicine. During this visit, the physician conducted a comprehensive medical assessment to confirm eligibility, reassessing the inclusion and exclusion criteria. Additionally, health risks were evaluated using the HeartScore2 calculator. Participants were also provided with detailed information regarding the study protocol and gave written consent.
  ○ **Visit 2**: During the second visit, participants worked with a physical activity trainer to assess their physical capacity. This assessment was based on the Cooper endurance test, where participants completed a 12 min run or walk on a treadmill, tailored to their initial capabilities [17]. Participants also reported the severity of any back pain using a visual analog scale (0–10) and underwent fitness tests, including the Thomayer (fingers-to-floor) test and a core stability test (plank).
  ○ **Visit 3**: In this visit with a nurse trained in health prevention and anthropometric assessment, participants underwent comprehensive anthropometric measurements, including height, body weight, body mass index (BMI) calculation, waist–hip ratio, and body composition analysis via bioimpedance using a Tanita scale (www.tanita.com, accessed on 18 September 2024) [18,19]. Blood pressure and heart rate were measured after a period of rest, and an electrocardiogram (ECG) was performed to assess cardiac health.

### 3.2. Physical Activity Stage

The physical activity intervention, lasting six months, was supported by qualified trainers and included both group and individual exercise sessions tailored to each participant’s capabilities and fitness levels. Exercise intensity was aligned with the World Health Organization (WHO) recommendations, promoting a minimum of 150–300 min of moderate-intensity exercise or 75–150 min of high-intensity exercise per week, or a combination of both [20]. Progress throughout the exercise program was closely monitored, and individual exercise plans were adjusted as needed by the physical activity trainer. Additionally, participants had the option to use the MyZone wristband (https://www.myzone.org/ accessed on 18 September 2024) to monitor their physical activity. This device enabled online tracking of training parameters, including duration, intensity, and type of activity, as well as physiological metrics such as heart rate and energy expenditure.

### 3.3. Summary Stage

At the conclusion of the six-month physical activity program, participants underwent a final assessment to evaluate the impact of the intervention. This included:

1. **Assessment of Physical Capacity**: A physical activity trainer conducted follow-up assessments using the Cooper test and fitness evaluations, along with an assessment of back pain severity.
2. **Nurse Visit**: A final visit with a nurse mirrored the initial assessment, encompassing the same range of measurements and tests to ensure consistency in data collection and to assess changes over the intervention period. Measurements, including body composition assessment, were performed in fasting participants on similar morning hours as in the initial visit in order to minimize variation.

This comprehensive methodological approach aimed to provide robust data on the effects of a structured physical activity program on body composition and health risk factors in the target population.

### 3.4. Statistical Methods

Descriptive statistics for numerical variables were presented as the number of non-empty observations, mean, standard deviation, median, minimum, and maximum; for categorical variables, they were presented as the number of occurrences and percentage of individual categories.

Univariate comparisons between independent groups of patients (division by gender, division into age groups 30+, 40+, and 50+, and division by baseline BMI below/above 30) were performed using the Fisher test or Pearson’s chi-square test (depending on the size of the compared groups) for categorical variables and using the Wilcoxon or Kruskal–Wallis rank sum test for numerical variables.

Comparisons between baseline and follow-up values were performed using McNemar’s tests (categorical variables) and paired Student’s *t*-test or Wilcoxon test (continuous variables, depending on the normality of the distribution tested using the Shapiro–Wilk test). These comparisons were made overall and for subgroups according to gender, age, and baseline BMI. For indicators whose reference values are gender different (body composition measurements), comparisons were carried out separately for women and men, divided into subgroups according to age and baseline BMI.

### 3.5. Ethical Aspects

The study was conducted as a research experiment, in accordance with the requirements of the Act of 5 December 1996 on the professions of doctor and dentist. [Journal of Laws 2023.0.1516], chapter 4. Medical experiment.

The study protocol, information materials for patients, including a template of informed consent to participate in the project, study insurance, and other required documents have received a positive opinion from the Bioethical Committees at the Regional Chamber of Physicians in Warsaw (No. Z/23477/1418/22 of 9 January 2023) and the Lower Silesian Chamber of Physicians in Wrocław (No. 06/BOBD/2022 of 14 December 2022).

Each participant included in the study gave written consent after being informed about the goals and course of the study, as well as potential risks and benefits. In addition, participants consented to the processing of personal data in accordance with the requirements of the General Data Protection Regulation (GDPR).

Analysis of factors influencing changes of the percentage of fat mass (%FM) and percentage of muscle mass (%MM) was performed with univariate linear regression models, overall and separately for women and men.

## 4. Results

The study participants were appropriately selected, with a gender distribution of 70% men and 30% women, reflecting the higher health-risk profile observed in middle-aged men within this population (the mean age of the study group was 46.6 years). Both subgroups of overweight (BMI 26–29.9, *n* = 86) and obese (BMI 30–35, *n* = 80) individuals were well-represented, ensuring a balanced representation of participants. Detailed demographic characteristics of the study population are provided in Table 1.

The analysis of baseline body mass composition measures and age revealed a significant positive correlation with visceral fat rating in both men (r = 0.364, *p* < 0.001) and women (r = 0.420, *p* = 0.002). A comprehensive summary of all baseline correlation data is presented in Table 2.

Visceral fat rating was the only body composition measure that exhibited significant differences across age groups (30–39, 40–49, and over 50) at both baseline and after the six-month training period. At baseline, the mean visceral fat ratings (±SD) for men were 10.5 (3.0), 11.4 (2.7), and 13.0 (2.4) (*p* = 0.002), while after six months, the ratings were 9.6 (3.3), 10.8 (2.8), and 11.7 (3.0) (*p* = 0.04), respectively. For women, baseline ratings were 6.2 (0.8), 8.3 (1.8), and 9.1 (2.0) (*p* = 0.003), with post-training ratings of 6.4 (1.1), 7.2 (1.4), and 8.8 (1.2), respectively.

Following the six-month training, all participants demonstrated significant improvements in their Cooper test results, with positive changes observed across subgroups categorized by gender, initial BMI, and age (all comparisons *p* < 0.001). Notably, 93% of participants achieved higher Cooper test scores, with an average improvement of approximately 30%. However, men exhibited significantly greater progress (33.4%) compared to women (21.8%) (see Table 3).

The back pain index, measured on a visual analog scale (VAS) ranging from 1 (no pain) to 10 (most severe pain), demonstrated a significant reduction following the six-month training period for all participants, with mean scores of 2.1 (SD ± 2.3) compared to 0.9 (SD ± 1.6) (*p* < 0.001). This improvement was consistent across subgroups, including men and women as well as individuals with BMI < 30 and BMI ≥ 30.

Additionally, most physical fitness tests showed significant enhancements after the training. The fingers-to-floor test exhibited improvement in men (*p* < 0.001), although no significant change was observed in women (*p* = 0.607). In contrast, the plank test demonstrated improvement in the overall group (*p* < 0.001) and within both gender subgroups (*p* < 0.001).

### 4.1. BMI and Body Mass Composition

At baseline, the body mass index ranged from 27.8 to 32.2 in the overall study group, with a median of 29.8. In men, the baseline BMI ranged from 27.7 to 32.3 (median 29.6), while in women, it ranged from 28.6 to 31.6 (median 30.1).

A comparative analysis of initial and post-intervention BMI values revealed significant differences across the entire study population, as well as within subgroups categorized by gender and initial BMI (<30 and ≥30) (see Table 4). The average decrease in BMI of 0.62 (SD ± 1.35) was statistically significant for the overall population; however, no significant difference was observed between men and women (*p* = 0.898). Notably, the difference in BMI change after six months approached statistical significance between subjects with initial BMI < 30 and those with BMI ≥ 30 (*p* = 0.048).

After six months of intervention, all men demonstrated a statistically significant reduction in fat tissue mass (FM), irrespective of their initial BMI. Specifically, the mean FM value (SD) decreased from 24.65 (6.68) kg to 22.58 (7.36) kg (*p* < 0.001). For men with an initial BMI < 30, the mean FM value (SD) decreased from 19.78 (3.79) kg to 18.18 (4.46) kg (*p* < 0.001), while those with an initial BMI ≥ 30 experienced a reduction from 30.13 (4.69) kg to 26.88 (5.62) kg (*p* = 0.002).

In contrast, statistical significance for changes in FM was only observed in women with an initial BMI ≥ 30, where the FM (SD) decreased from 33.88 (5.60) kg to 31.81 (5.39) kg (*p* = 0.012). Additionally, a significant reduction in the percentage of body fat was noted among all men, with the mean percentage (SD) declining from 25.35 (4.32)% to 23.64 (5.69)% (*p* < 0.001). This decrease was consistent across different initial BMI categories. For men with a baseline BMI < 30, the mean percentage (SD) of body fat decreased from 22.34 (2.80)% to 20.83 (4.03)% (*p* = 0.02), whereas those with an initial BMI ≥ 30 saw a reduction from 28.75 (3.01)% to 26.88 (5.62)% (*p* = 0.009).

In the female participants, the overall change in the percentage of body fat was statistically insignificant, with values shifting from 36.83 (4.42)% to 36.25 (3.13)% (*p* = 0.297) in the entire group and from 34.57 (4.60)% to 34.88 (2.83)% (*p* = 0.742) in those with an initial BMI < 30. Conversely, women with an initial BMI ≥ 30 experienced a significant reduction in body fat percentage, decreasing from 39.01 (2.95)% to 37.57 (2.85)% (*p* = 0.023).

The visceral fat rating (VFR) showed a statistically significant decrease in all male participants, regardless of initial BMI. Similarly, the visceral fat tissue index decreased significantly in the overall female group and, specifically, in the subgroup with BMI ≥ 30. Detailed data are provided in Table 5.

Visceral fat rating decreased significantly across all three male age subgroups (30–39, 40–49, and 50+) following the six-month training period, with mean (SD) values showing the following changes: 10.5 (3.02) to 9.6 (3.26) (*p* = 0.034) for ages 30–39; 11.4 (2.67) to 10.8 (2.79) (*p* = 0.006) for ages 40–49; and 13.0 (2.36) to 11.7 (2.96) (*p* < 0.001) for ages 50+. Due to small sample sizes, the female age subgroups were not suitable for further age-related analysis.

While nominal changes in muscle tissue mass (MM) did not reach statistical significance across the groups of men and women, all male participants demonstrated a significant increase in the percentage of muscle tissue (%MM) after six months of exercise. The mean percentage (SD) increased from 70.92 (4.09)% to 72.79 (6.01)% (*p* < 0.001), and this improvement was consistent regardless of initial BMI. Specifically, in men with a baseline BMI < 30, the mean %MM increased from 73.79 (2.62)% to 75.66 (5.14)% (*p* = 0.004), while in men with an initial BMI ≥ 30, it rose from 67.69 (2.83)% to 69.55 (5.25)% (*p* = 0.007).

In contrast, changes in the percentage of muscle tissue among women were statistically insignificant, with the exception of those with an initial BMI ≥ 30, where %MM increased significantly from 57.90 (2.84)% to 59.24 (2.70)% (*p* = 0.023). Additionally, changes in lean tissue mass and body water content did not achieve statistical significance in any of the analyzed subgroups.

### 4.2. Exploratory Analysis %FM and %MM

Training duration in 80–89% HRmax significantly correlated with a decrease in fat mass percentage (%FM) among males, with each additional hour of training leading to a reduction of 0.16 percentage points in %FM. Furthermore, training in the 70–79% HRmax zone significantly promoted increases in muscle mass percentage (%MM), with each hour of training associated with a 0.10 percentage point rise in %MM. Additionally, training duration in the 80–89% HRmax zone also significantly enhanced %MM, with every training hour linked to a 0.22 percentage point increase.

In contrast, no statistically significant effects were observed in the simple regression models for %FM and %MM in females, as detailed in Table 6 and Table 7.

## 5. Discussion

The results of this study confirmed that in overweight and obese middle-aged population between 30 and 60 years old, several body composition elements are correlated with age and exhibit negative changes from younger to older age groups, with the visceral fat rating showing especially marked growth. These changes might be at least partially reversed by physical activity, showing, after six months, positive effects on BMI and the percent of fat mass (decrease) and percent of muscle mass (increase), and, in particular, on visceral fat reduction. Positive body mass composition effects were associated with high-intensity training with heart rate above 70% of maximum for the age in men but not in women.

### 5.1. Fitness and Health Improvements

The six-month exercise cycle markedly improved the fitness and physical performance of the study participants, as evidenced by a significant improvement in the results in the Cooper test. There was improvement in 93% of participants, on average, by approximately 30%, but significantly greater progress was noted in men (33.4%) compared to women (21.8%). Overweight subjects achieved a better percentage improvement in the Cooper test after six months than obese participants, but this difference was not significant, so it should be assumed that the improvement in the Cooper test results did not depend on the initial BMI value. Interestingly, although the Cooper endurance test has been recommended as a simple method for checking fitness level, little was reported on Cooper test result changes after the physical training program.

A meaningful improvement in other fitness tests (plank, finger-to-floor) and a significant decrease in the back pain scale were also observed. Apart from the general health risk, improving these fitness parameters is also of great importance for the professionally active population, among which, one of the main negative phenomena is absenteeism and productivity problems related to musculoskeletal disorders resulting from a sedentary lifestyle [21]. Altogether, the results of this study confirmed that, within a few months of moderate to vigorous physical activity, marked fitness improvements can be achieved with relatively simple training regimen. This is in line with previous reports that showed the positive effects of physical activity, such as improved fitness or weight loss, observed even after relatively short training time i.e., three months [22,23]; but, certainly, longer and regular training is required to achieve full benefits. It has been shown in large cohort studies that physical activity, when performed on the level recommended by guidelines, was related to a markedly diminished, by 30–40%, mortality risk [24,25].

The physical activity protocol in this study, which used some support and moderation by trained coaches with access to supervised training in fitness centers, is relatively easy to be implemented on a large scale. In addition, based on our and others’ experience, this can bring better results, especially when combined with wearable device that monitors activity time and heart rate, than just individual exercise [26]. Thus, such an approach might be recommended for public systems and for wellness programs offered or subsidized by employers. The latter might be of particular importance, as evidence shows that, for the best health results and prevention of age-related problems, one should engage in activity early, ideally, before age 50–55 [27,28]. So, the physical activity target age should be 40–50 years old, or even younger, exactly as the population in the present study indicates.

### 5.2. Muscle Mass, Fat Mass, and Visceral Fat Effects

There is vast evidence that overweight and obesity strongly increase health risk and the development of many diseases, including diabetes, cardiovascular disorders, etc. However, relatively few studies have examined the association between body composition and health risk or mortality. The results were mixed, but in general, suggested that high fat mass (especially higher range) and low lean body mass (especially lower range) were associated with increased risk of mortality [29]. Among body composition elements, high visceral fat was associated with increased cardiovascular risk in women [30] and was a strong, independent predictor of all-cause mortality in men [31]. Visceral fat mass might be high and increase health risk even in people with normal body weight and BMI [32], which suggests that it should be considered as an additional risk factor and measured by means of waist–hip ratio or, better, by a method directly assessing visceral fat content.

In this study, neither BMI nor fat mass but visceral fat rating (VFR) was correlated with age in both sexes, indicating a growing risk independent of other body components. After the six-month training period, besides a significant reduction in body fat mass and the percent of body fat, VFR decreased by 7.4% in men and by 6.6% in women. It was the strongest change among all measured parameters, which provides evidence of the highly positive impact of physical activity, beyond changes in BMI and other factors. These results support observations that even after relatively short time periods of a few weeks of inactivity and sedentary behavior, body and visceral fat mass increases, which could be reversed by physical training [33,34].

Studies suggest that muscle mass decreases by about 3 to 8 percent per decade after age 30 and at higher rates after age 60 [35]. Low muscle mass was associated with cardiovascular and mortality risk in general population [36], and in middle-aged people with obesity [37]. Losing muscle strength may have a significant negative effect on body balance and other health consequences not only in elderly life, but also for being active and productive at work. In the present analysis, muscle mass was inversely correlated with age in women, but not in men. After the six-month training period, the percentage of muscle mass, but not of nominal muscle mass, increased significantly in men and the subgroup of women with obesity, which suggests another additional positive change due to physical activity.

### 5.3. High-Intensity Training

Age-related body changes are further exacerbated by a sedentary lifestyle and can be, in part, prevented by the maintenance of activity with aging [38]. Physical fitness markers, such as strength and cardiorespiratory fitness, were the strongest predictors for body composition rather than just physical activity [15] The results of this study are consistent with findings that physical activity may, at least partially, reverse negative changes in body mass composition related to aging by increasing muscle mass and decreasing fat mass [39].

In this study, interesting results came from exploratory analysis regression models for men, where the percentage of muscle mass has been found to be significantly dependent on time spent in high-intensity training with 70–79% and 80–89% HRmax, and the percent fat mass inversely related with training time with 80–90% HRmax. This is a relatively new finding, in agreement with recent evidence that high-intensity interval training (HIIT) induces numerous physiological adaptations that improve exercise capacity (maximal oxygen uptake, endurance, etc.) and metabolic health in both clinical and healthy populations [40].

In an up-to-date review on HIIT’s influence on body composition, it was concluded that it promotes weight loss, reduces BMI, and decreases the content of adipose tissue, including visceral fat [41]. However, it has been also suggested that further analyses are needed to systematize knowledge of the benefits of different HIIT protocols [42]. The present results add some insight, showing that vigorous exercise within high heart rate zones positively affects key body mass components including the percent of fat and muscle mass. This might support proposals for modification to physical activity recommendations to include not only training time but also periods with higher effort as optimal for health benefits.

The findings of the clear benefits of high-intensity interval training or, as proved in this analysis, more time spent in higher heart rate zones, can also be helpful in promoting sport activities. In Poland, where physical activities are quite rare in the population, surveys show that one of the reasons for non-activity is a lack of time [13]. The presented data provide evidence that the best outcome can be reached not by lengthy exercise, but by shorter sessions with higher intensity protocols. On top of that, it was reported that high-intensity effort, even for a short time, supports the release of mood-boosting brain chemicals such as endorphins [43], which should increase positive connotations with the sport activity, encouraging individuals to continue regular use.

### 5.4. Limitations of the Study

There are several factors which may limit the conclusions of our study. First of all, comparisons were only recorded between the initial and six-month results in intervention participants, but there was no control group. However, data from larger medical database of populations with similar characteristics (gender, age, weight, etc.) as the study group revealed that no changes in BMI or other parameters occurred during same six-month time period. Positive changes in fitness and body composition should also be verified in longer observational studies to confirm their longitudinal benefit. Additionally, more detailed information on the Cooper test conditions (such as speed, slope, and heart rate control) and the specific workloads for different age groups (30–39, 40–49, etc.) could provide further clarity. The body composition scales used were applied consistently, though specific calibration details were not outlined. The optional use of wristbands was not systematically analyzed, which may limit the interpretation of physical activity adherence. Finally, while our focus was on fitness and body composition, this study did not examine nutrition or lipid profile changes, which may be an area for further research.

## 6. Conclusions

The findings from this study underscore the significant impact of a six-month supervised physical activity program on mitigating health risks and improving body composition among middle-aged individuals with overweight or obesity. Participants demonstrated marked improvements in key metrics, including visceral fat rating, fat mass, and muscle mass, particularly among men, indicating that structured exercise interventions can effectively address obesity-related health issues.

Furthermore, the analysis revealed that training at higher intensities (70–89% HRmax) yielded greater reductions in fat mass and increases in muscle mass, suggesting that optimizing exercise intensity could enhance the efficacy of physical activity programs. These results advocate for the integration of tailored, intensity-focused exercise regimens into public health strategies aimed at reducing obesity and its associated health risks.

In light of these outcomes, it is recommended that healthcare providers and fitness professionals develop and promote supervised exercise interventions that not only adhere to established guidelines but also emphasize intensity to maximize health benefits. Additionally, further research should investigate the long-term effects of such interventions and explore individualized approaches to accommodate diverse populations and their unique health profiles.

## Figures and Tables

**Table 1:** Baseline demographic and anthropometric measurements of the studied population.

Data in Gender Groups	Total, N = 166	Males, N = 117	Females, N = 49	*p*-Value ^1^
Age				0.009
Mean (SD)	46.6 (7.2)	45.6 (6.7)	49.0 (7.8)	
Median [IQR]	47.0 [41.2, 51.8]	46.0 [41.0, 50.0]	50.0 [43.0, 55.0]	
Minimum, Maximum	30, 66	30, 64	31, 66	
Height				<0.001
Mean (SD)	174.4 (8.7)	178.5 (5.9)	164.5 (6.0)	
Median [IQR]	176.0 [169.0, 181.0]	179.0 [175.0, 182.0]	165.0 [161.0, 168.0]	
Minimum, Maximum	150, 194	160, 194	150, 178	
Weight				<0.001
Mean (SD)	91.7 (12.8)	95.7 (11.8)	81.9 (9.6)	
Median [IQR]	90.0 [82.0, 101.0]	94.0 [87.0, 106.0]	82.0 [76.0, 86.0]	
Minimum, Maximum	64, 127	69, 127	64, 107	
BMI				0.420
Mean (SD)	30.05 (2.77)	29.98 (2.85)	30.24 (2.60)	
Median [IQR]	29.80 [27.80, 32.22]	29.60 [27.70, 32.30]	30.10 [28.60, 31.60]	
Minimum, Maximum	24.4, 37.8	24.4, 37.8	25.0, 36.3	
BMI Group, N (%)				0.637
BMI below 30	86 (52%)	62 (53%)	24 (49%)	
BMI 30 and above	80 (48%)	55 (47%)	25 (51%)	
Age Group, N (%)				0.031
Age 30–39	27 (16%)	22 (19%)	5 (10%)	
Age 40–49	79 (48%)	60 (51%)	19 (39%)	
Age 50+	60 (36%)	35 (30%)	25 (51%)	

^1^ Wilcoxon rank sum test; Pearson’s Chi-squared test, comparison males vs. females.

**Table 2:** Baseline correlations of body mass composition metrics with age (separately for males and females due to differences in body mass norms).

	Males, N = 117		Females, N = 49	
Body Composition Metrics	Correlation Coefficient ^1^	*p* Value	Correlation Coefficient ^1^	*p*-Value
BMI	−0.043	NS	−0.144	NS
Fat mass (kg)	−0.041	NS	−0.101	NS
%FM	0.003	NS	0.109	NS
Visceral fat rating	0.364	<0.001	0.420	0.002
TBW (kg)	−0.184	0.047	−0.384	0.006
FFM (kg)	−0.057	NS	−0.379	0.007
Muscle mass (kg)	−0.100	NS	−0.379	0.007
%MM	0.003	NS	−0.108	NS

^1^ Pearson correlation.

**Table 3:** Cooper test results at baseline and after 6-month training according to gender, initial BMI value, and age group *.

Analyzed Subgroups	Cooper Test Results—Baseline			Cooper Test Results—6 Months			*p*-Value ^1^
	Mean (SD)	Median [IQR]	Minimum, Maximum	Mean (SD)	Median [IQR]	Minimum, Maximum	
All participants (N = 150–105)	1459.4 (391.9)	1352.0 [1192.5, 1680.0]	618, 2530	1842.4 (420.0)	1820.0 [1520.0, 2200.0]	1010, 2720	<0.001
Males (N = 103–73)	1561.4 (407.2)	1470.0 [1230.0, 1810.0]	800, 2530	1999.1 (367.1)	2017.0 [1780.0, 2270.0]	1160, 2720	<0.001
Females (N = 47–32)	1235.8 (235.6)	1190.0 [1095.0, 1340.0]	618, 1830	1484.8 (298.1)	1400.0 [1290.5, 1602.5]	1010, 2330	<0.001
Initial BMI < 30 (N = 77–58)	1543.7 (456.0)	1411.0 [1180.0, 1850.0]	820, 2530	1959.9 (420.9)	1925.0 [1642.5, 2302.5]	1180, 2720	<0.001
Initial BMI ≥ 30 (N = 73–47)	1370.4 (287.6)	1330.0 [1200.0, 1550.0]	618, 2110	1697.3 (374.6)	1641.0 [1389.0, 2011.0]	1010, 2410	<0.001
Age 30–39 (N = 25–24)	1672.8 (448.9)	1750.0 [1230.0, 2060.0]	1050, 2530	1974.4 (426.0)	1985.0 [1835.0, 2164.0]	1167, 2800	<0.001
Age 40–49 (N = 68–69)	1502.2 (355.8)	1430.0 [1262.2, 1685.0]	618, 2500	1847.7 (383.3)	1830.0 [1556.0, 2120.0]	1002, 2750	<0.001
Age 50+ (N = 57–51)	1314.7 (356.4)	1200.0 [1090.0, 1505.0]	800, 2500	1626.2 (373.1)	1540.0 [1357.5, 1810.0]	1125, 2600	<0.001

^1^ Paired *t*-test. * (in brackets are numbers of available results for each subgroup and measurement).

**Table 4:** Baseline BMI and after 6-month training in the whole studied group, and in subgroups according to gender, initial BMI, and age.

Analyzed Groups	BMI—Baseline			BMI—6 Months Training			*p*-Value ^1^
	Mean (SD)	Median [IQR]	Minimum, Maximum	Mean (SD)	Median [IQR]	Minimum, Maximum	
All subjects (N = 166)	30.05 (2.78)	29.76 [27.77, 32.20]	24.4, 37.8	29.43 (2.87)	29.14 [27.12, 31.28]	24.4, 37.0	<0.001
Males (N = 117)	29.97 (2.85)	29.63 [27.68, 32.27]	24.4, 37.8	29.36 (2.92)	29.05 [26.99, 31.31]	24.4, 35.6	<0.001
Females (N = 49)	30.24 (2.61)	30.11 [28.63, 31.62]	25.0, 36.3	29.60 (2.75)	29.37 [27.83, 31.12]	24.6, 37.0	0.005
Initial BMI < 30 (N = 86)	27.81 (1.26)	27.77 [26.84, 28.73]	24.4, 29.8	27.39 (1.55)	27.15 [26.24, 28.41]	24.4, 30.8	<0.001
Initial BMI ≥ 30 (N = 80)	32.45 (1.76)	32.38 [30.98, 33.66]	30.0, 37.8	31.62 (2.28)	31.31 [29.76, 33.17]	24.6, 37.0	<0.001
Age 30–39 (N = 27)	30.29 (3.13)	30.12 [27.77, 32.43]	25.9, 37.8	29.90 (3.42)	28.73 [27.42, 33.47]	25.1, 35.6	0.167
Age 40–49 (N = 79)	30.07 (2.68)	29.76 [27.76, 32.48]	24.4, 36.3	29.31 (2.73)	29.01 [27.12, 31.13]	24.4, 35.5	<0.001
Age 50+ (N = 60)	29.92 (2.78)	29.75 [27.97, 31.72]	25.0, 36.5	29.38 (2.80)	29.38 [27.09, 31.25]	24.6, 37.0	0.004

^1^ Paired *t*-test.

**Table 5:** Baseline visceral fat rating (VFR) and after 6-month training in the male and female subgroups and according to initial BMI.

Analyzed Groups	VFR—Baseline			VFR—6 Months Training			*p*-Value ^1^
	Mean (SD)	Median [IQR]	Minimum, Maximum	Mean (SD)	Median [IQR]	Minimum, Maximum	
**Males all (N = 117)**	11.72 (2.78)	12.00 [9.00, 13.00]	6.0, 19.0	10.85 (2.99)	10.00 [9.00, 13.00]	4.0, 16.0	<0.001
**Males BMI < 30 ** **(N = 62)**	9.81 (1.77)	10.00 [9.00, 11.00]	6.0, 13.0	9.02 (1.96)	9.00 [8.00, 10.00]	5.0, 14.0	<0.001
**Males BMI ≥ 30 ** **(N = 55)**	13.87 (2.05)	14.00 [13.00, 15.00]	8.0, 19.0	12.89 (2.60)	13.00 [12.00, 15.00]	4.0, 16.0	0.003
**Females all (N = 49)**	8.50 (2.00)	8.00 [7.00, 10.00]	4.0, 13.0	7.94 (1.56)	8.00 [7.00, 9.00]	5.0, 12.0	0.019
**Females BMI < 30** **(N = 24)**	7.48 (1.41)	8.00 [7.00, 8.00]	4.0, 10.0	7.29 (1.43)	7.00 [6.00, 8.00]	5.0, 10.0	0.601
**Females BMI ≥ 30** **(N = 25)**	9.44 (2.02)	9.00 [8.00, 11.00]	6.0, 13.0	8.56 (1.45)	8.00 [8.00, 9.00]	6.0, 12.0	0.014

^1^ Paired *t*-test.

**Table 6:** Simple linear regression models for percent fat mass (%FM) in males.

Characteristic	Beta	95% CI ^1^	*p*-Value
Age subgroup			
30–39	—	—	
40–49	1.2	−0.91, 3.3	0.265
50+	−1.6	−3.9, 0.65	0.159
BMI subgroup			
BMI < 30	—	—	
BMI ≥ 30	−0.37	−2.0, 1.2	0.648
Total calories burned [thousands kcal]	−0.02	−0.04, 0.00	0.088
Training duration in 0–49% HRmax [hours]	0.00	−0.01, 0.01	0.949
Training duration in 50–59% HRmax [hours]	−0.01	−0.03, 0.02	0.662
Training duration in 60–69% HRmax [hours]	−0.02	−0.06, 0.02	0.231
Training duration in 70–79% HRmax [hours]	−0.06	−0.13, 0.01	0.074
Training duration in 80–89% HRmax [hours]	−0.16	−0.28, −0.05	0.007
Training duration in 90–100% HRmax [hours]	−0.28	−0.79, 0.24	0.290
Total training duration [hours]	0.00	−0.01, 0.00	0.461
Max HR	−0.01	−0.08, 0.06	0.813
Mean training effort [as % HRmax]	−0.14	−0.32, 0.03	0.106

^1^ CI = Confidence Interval.

**Table 7:** Simple linear regression models for percent muscle mass (%MM) in males.

Characteristic	Beta	95% CI ^1^	*p*-Value
Age subgroup			
30–39	—	—	
40–49	−0.60	−2.9, 1.7	0.598
50+	1.3	−1.1, 3.8	0.284
BMI subgroup			
BMI < 30	—	—	
BMI ≥ 30	0.09	−1.6, 1.8	0.916
Total calories burned [thousands kcal]	0.03	0.00, 0.05	0.027
Training duration in 0–49% HRmax [hours]	0.00	−0.01, 0.01	0.979
Training duration in 50–59% HRmax [hours]	0.01	−0.02, 0.03	0.607
Training duration in 60–69% HRmax [hours]	0.04	0.00, 0.08	0.070
Training duration in 70–79% HRmax [hours]	0.10	0.02, 0.18	0.015
Training duration in 80–89% HRmax [hours]	0.22	0.09, 0.35	0.001
Training duration in 90–100% HRmax [hours]	0.57	−0.01, 1.1	0.054
Total training duration [hours]	0.00	0.00, 0.01	0.356
Max HR	0.04	−0.04, 0.12	0.314
Mean training effort [as % HRmax]	0.18	−0.02, 0.38	0.079

^1^ CI = Confidence Interval.

## Data Availability

All data are available from the corresponding author.
